# De novo transcriptome assembly and population genetic analyses of an important coastal shrub, *Apocynum venetum* L

**DOI:** 10.1186/s12870-020-02626-7

**Published:** 2020-09-03

**Authors:** Na Yuan, Mimi Li, Chunlin Jia

**Affiliations:** 1 Institute of Crop Germplasm and Biotechnology, Provincial Key Laboratory of Agrobiology, Jiangsu Academy of Agricultural Sciences, Nanjing, China; 2 Institute of Botany, Jiangsu Province and Chinese Academy of Sciences, Nanjing, China; 3 Institute of Agricultural and Sustainable Development, Shandong Academy of Agricultural Sciences, Jinan, China

**Keywords:** *Apocynum venetum* L., Transcriptome, EST-SSR marker, Population genetics, Coastal wetland

## Abstract

**Background:**

*Apocynum venetum* L. is an important medicinal plant that is mainly distributed in the coastal areas and northwest of China. In addition to its high medical and economic value, its adaptation to saline-alkali and coastal saline lands makes *A. venetum* an ideal candidate for use in vegetation restoration. To date, the study of *A. venetum* has been limited in the northwest region of China, little attention has been paid to the genetic diversity and population structure of *A. venetum* populations in the coastal region. Here, we performed transcriptome sequencing of total RNA from *A. venetum* leaves and developed efficient expressed sequence tag-simple sequence repeat (EST-SSR) markers for analyzing the genetic diversity and population structure of *A. venetum* in the coastal region.

**Results:**

A total of 86,890 unigenes were generated after de novo assembly, and 68,751 of which were successfully annotated by searching against seven protein databases. Furthermore, 14,072 EST-SSR loci were detected and 10,243 primer pairs were successfully designed from these loci. One hundred primer pairs were randomly selected and synthesized, twelve primer pairs were identified as highly polymorphic and further used for population genetic analysis. Population genetic analyses showed that *A. venetum* exhibited low level of genetic diversity (mean alleles per locus, *N*_A_ = 3.3; mean expected heterozygosity, *H*_E_ = 0.342) and moderate level of genetic differentiation among the populations (genetic differentiation index, *F*_ST_ = 0.032–0.220) in the coastal region. Although the contemporary (mean *m*_c_ = 0.056) and historical (mean *m*_h_ = 0.106) migration rates among the six *A. venetum* populations were moderate, a decreasing trend over the last few generations was detected. Bayesian structure analysis clustered six populations into two major groups, and genetic bottlenecks were found to have occurred in two populations (QG, BH).

**Conclusions:**

Using novel EST-SSR markers, we evaluated the genetic variation of *A. venetum* in the coastal region and determined conservation priorities based on these findings. The large dataset of unigenes and SSRs identified in our study, combining samples from a broader range, will support further research on the conservation and evolution of this important coastal plant and its related species.

## Background

Coastal habitats, which are located in the transition zone between terrestrial ecosystems and marine ecosystems, are characterized by unique, complex ecosystems with high ecological value [1]. Their unique environmental features, including poor soil structure with low water infiltration and poor drainage, a high salt content and PH level, give rise to unique plant diversity and many specialist species [2]. However, due to the rapid urbanization process, coastal habitats have been particularly affected by both land transformation and mass tourism, leading to the severe disturbance and loss of natural habitats [3]. Many coastal plants are adapted to specific costal environmental conditions and are thus highly vulnerable to habitat destruction [4–6]. Therefore, there is an urgent need to evaluate and protect the irreplaceable, vulnerable biodiversity found in the coastal zones, especially in the face of continuing anthropogenic pressure.

The coastal area of Jiangsu Province accounts for one quarter of the total coastal area in China, which mainly falls within Yancheng City [7]. The coastline of Yancheng City’s is 582 km long, and the beach area covers 6.83 million hm^2^, with an annual growth rate of 10,000 hm^2^ [7]. The Yancheng coastal region not only provides precious land resources, but is also an important wetland nature reserve. According to the plant surveys, there are 688 kinds of vascular plants belonging to 391 genera and 114 families in the Yancheng tidal flat wetland [8]. Multiple large-scale beach reclamation projects have been conducted in Yancheng city since 1949, and the natural habitats of this region have been significantly modified [7]. Many studies have shown that anthropogenic habitat alterations can affect both global biodiversity and genetic diversity, which jeopardizes the long-term survival of species and increases their risk of extinction [9–13]. There are many valuable plant resources, such as *Limonium sinense* (Girard) Kuntze, *Apocynum venetum*, *Tamarix chinensis* Lour., *Tournefortia sibirica*, and *Salicornia europaea* L. etc. in the Yancheng tidal flat, which are not only tolerant to salt stress but also present high medical and economic values [8]. Whether the genetic diversity and genetic structure of coastal plant populations have been affected by coastal habitat changes has seldom been evaluated.

*Apocynum venetum* L*.* is a perennial shrub that is specifically distributed in the coastal region of Jiangsu Province. It has also been referred to as “Luobuma” since it was first discovered on the Luopu plains of Xinjiang Province in the 1950s [14]. *A. venetum* not only provides precious fiber and nectar resources but is also used as an important medicinal plant for treating hypertension and hyperlipidemia. Its high stress resistance to high salt contents and poor soils also contribute to its great ecological value. To date, studies of *A. venetum* have mainly focused on its medicinal effects and physiological characteristics such as photosynthesis and water absorption [15–17]. However, few studies have examined the genetic diversity and genetic structure of natural *A. venetum* populations. To better protect and utilize this important plant species in the Yancheng coastal region, there is an urgent need to evaluate the genetic diversity and population structure of natural *A. venetum* populations.

Simple sequence repeat (SSR) markers have been widely used in population genetic analysis and molecular marker-assisted breeding because of their high polymorphism, repeatability and codominant inheritance [18, 19]. A number of transcriptome analyses have been conducted on *A. venetum* [20–22], while SSR markers have not yet been developed and used to evaluate the genetic diversity of natural *A. venetum* populations yet. Using RAPD (Random Amplification Polymorphic DNA) and AFLP (Amplified Fragment Length Polymorphism) markers, researchers have detected moderate to high levels of genetic diversity in *A. venetum* populations from Xinjiang and Inner Mongolia regions [23, 24]. Except in the arid region, there has been no study that has evaluated the genetic diversity of *A. venetum* populations in the coastal regions. Therefore, in this study, we conducted comprehensive transcriptome sequencing of *A. venetum* from the coastal region of Jiangsu Province using the Illumina sequencing platform. After data assembly and annotation, we developed a set of novel EST-SSR markers from the unigenes. By using these informative markers, we successfully evaluated the genetic diversity, population structure and demographic changes in six populations across the natural distribution of *A. venetum* in the Yancheng coastal region. We expect that the genetic information identified in this study will facilitate the management and conservation of natural *A. venetum* populations in the future.

## Results

### Assembly of *A. venetum* transcriptome data from Illumina sequencing

Transcriptome sequencing generated 46,408,308 reads, totalling approximately 6.96 Gb for *A. venetum* in this study. After stringent quality filtering, 45,760,331 (98.6%) high-quality reads were obtained, exhibiting 98.31% Q20 bases and a GC value of 46.93% (Table 1). A total of 86,890 unigenes were successfully assembled using the Trinity software, with a mean length of 1767 bp and an N50 of 2580 bp. Among all the assembled unigenes, 3119 of which (approximately 3.58%) were less than 300 bp, and 14,657 unigenes (16.85%) were longer than 3000 bp, whereas most of the unigenes (69,204) (79.56%) ranged from 300 to 3000 bp (Additional file 1: Fig. S1A). The number of reads mapped to each unigene analysis revealed that 13,065 unigenes (about 47.90%) and 5506 unigenes (approximately 20.19%) consist of more than 100 and 1000 reads each, respectively, only a few unigenes (3.3%) were derived from less than 10 reads (Additional file 1: Fig. S1B).

**Table 1:** Sequencing, assembly, and annotation results of *A. venetum* transcriptome

Description	Number
1. Raw sequences and Assembly statistics	
High-quality reads	45,760,331
Total nucleotides of high-quality reads (bp)	16,326,427,500
Q20 percentage	98.31%
GC percentage of high-quality reads	46.93%
Number of unigenes	86,980
Range of unigenes length (bp)	201–17,334
N50 length of unigenes (bp)	2580
2. Bioinformatics annotations of unigenes	
Gene annotated against NR(%)	63,975 (73.55)
Gene annotated against NT(%)	48,088 (55.28)
Gene annotated against KO(%)	28,505 (32.77)
Gene annotated against SwissProt(%)	53,436 (61.43)
Gene annotated against PFAM(%)	51,451 (59.15)
Gene annotated against GO(%)	51,733 (59.47)
Gene annotated against KOG(%)	23,428 (26.93)
Gene annotated against all Databases(%)	13,207 (15.18)
Gene annotated against at least one Database(%)	68,751 (79.04)
Gene Unannotated	18,229 (20.96)

### Functional annotation

Using NCBI BLAST tools, all assembled unigenes were searched against Nr, Nt, SwissProt, Pfam, GO, KO and KOG databases for functional annotation. Among the 86,980 unigenes, the greatest number of matches were annotated in the Nr database (63,975 unigenes, 73.55% of all unigenes), followed by the Swiss-Prot (53,436, 61.43%), GO (51,733, 59.47%) and Pfam (51,451, 59.15%) databases (Table 1). In total, 68,751 (79.04%) unigenes exhibited homologous matches in at least one database and 13,207 (15.18%) unigenes were annotated in all seven databases (Table 1).

### Functional classification by GO and KOG

**Fig. 1:**
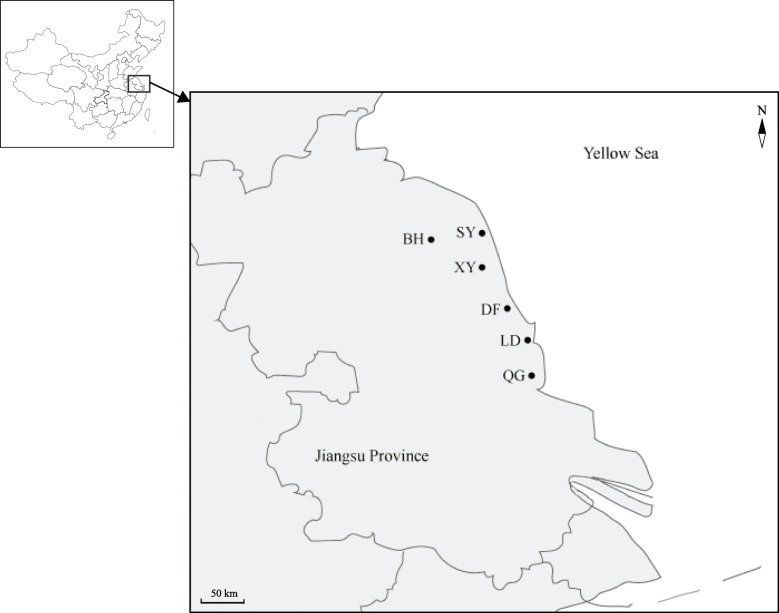
Functional classification of Gene Ontology (GO) for assembled unigenes of *A. venetum*. A total of 51,733 unigenes were assigned into 54 functional groups of three GO categories

To further evaluate the functions of these unigenes, we used GO assignments to annotate and analyze each unigene. A total of 51,733 unigenes were assigned to 54 functional subgroups. Among the three ontology categories, the largest was biological process (47.6%), followed by cellular component (29.5%) and molecular function (22.9%) (Fig. 1). In the biological process group, the most frequent category was cellular process (22.04%), followed by metabolic process (20.99%). Cell (19.40%) and cell part (19.40%) were the most highly represented groups in the cellular component category. For the molecular function category, binding (45.49%) and catalytic activity (38.16%) accounted for the greatest proportions. Then we submitted all the assembled unigenes to the KOG database for further functional prediction and classification. Among the 25 KOG categories, posttranslational modification, protein turnover and chaperones consist the largest group (13.36%), general function prediction only (12.71%) and translation, ribosomal structure and biogenesis (10.60%) also showed high percentages (Additional file 2: Fig. S2).

### Functional classifications by KEGG

**Fig. 2:**
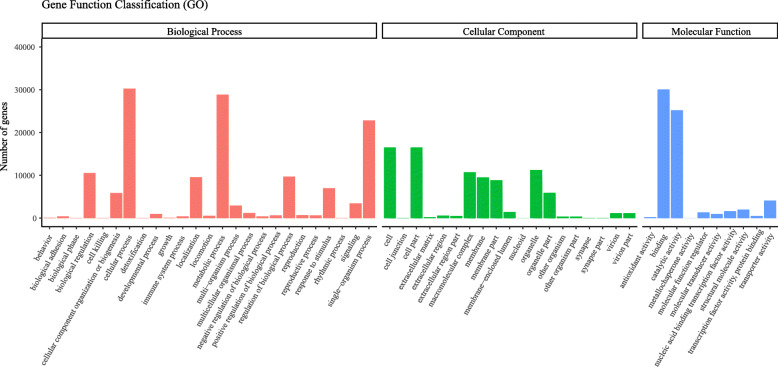
Functional classification of unigenes of *A. venetum* in KOG category. Total of 23,428 unigenes were assigned into 25 functional groups

A total of 28,505 unigenes were assigned to 130 KEGG pathways that belonged to five categories, namely, metabolic pathways (44.08%), genetic information processing (22.55%), cellular processes (4.5%), environmental information processing (3.25%) and organismal systems (2.89%) (Fig. 2). The majority of the unigene pathways were associated with translation (2847 unigenes), carbohydrate metabolism (2553 unigenes), folding, sorting and degradation (1941 unigenes).

### Development and characterization of SSR markers

In this study, a total of 14,072 SSRs with motifs ranging from di- to hexanucleotides were identified from 86,980 unigenes. Among all the SSR types, dinucleotide repeats accounts for the major proportion (9399, 66.8%), followed by trinucleotides (4328, 30.76%). Tetranucleotide (210, 1.49%), hexanucleotide (93, 0.66%) and pentanucleotide (42, 0.30%) repeats were very low-frequency types. The numbers of tandem repeats of these SSRs ranged from five to 36, and the most abundant repeat unit was six (3571, 25.38%), followed by five tandem repeats (2670, 18.97%) and seven tandem repeats (1950, 13.86%) (Table 2). Among the dinucleotide repeats, the most abundant motif type was AG/CT (34.50%), followed by AT/AT (23.39%) and AC/GT (8.66%). Among the trinucleotide repeats, AAG/CTT (8.99%) was the most frequent motif type, followed by AAT/ATT (6.07%). The remaining motif types accounted for 18.4% of these repeats (Table 2).

**Table 2:** The distribution of *A. venetum* EST-SSRs based on the number of repeat units

Number of repeat unit	Di-	Tri-	Tetra-	Penta-	Hexa	Total
5	0	2395	174	27	74	2670
6	2502	1008	28	14	19	3571
7	1487	456	7	0	0	1950
8	1151	209	1	1	0	1362
9	944	118	0	0	0	1062
10	639	91	0	0	0	730
11	545	11	0	0	0	556
12	448	13	0	0	0	461
13	255	0	0	0	0	255
14	345	12	0	0	0	357
≥15	1083	15	0	0	0	1098

Within the 14,072 SSRs, 10,243 primer pairs were successfully designed. We randomly selected 100 pairs from these primer pairs for amplification, and 35 were successfully amplified at expected sizes. Using twelve individuals from four *A. venetum* populations, these 35 primer pairs were applied to screen for polymorphism and twelve showed allelic polymorphism (Table 3). Using these 12 polymorphic EST-SSR markers, a total of 39 alleles were detected across the 103 samples from the six coastal *A. venetum* populations, with 2 to 5 alleles per locus. Using the Micro-Checker program, we found no evidence of null alleles and scoring errors in the dataset. Linkage disequilibrium analysis was performed between each pair of loci in each population. The results showed that 6 of 396 comparisons (*Av39* and *Av88* in population LD; *Av02* and *Av88* in population XY; *Av08* and *Av39*, *Av08* and *Av63*, *Av39* and *Av63*, *Av39* and *Av75* in population SY) were significant after Bonferroni correction (*P* = 0.00013). Given that there is no overlap between these six pairs of loci, we treated these 12 EST-SSR markers as independent loci in the following analyses. For all loci, the expected (*H*_E_) and observed heterozygosities (*H*_O_) ranged from 0.030 to 0.651 and from 0.030 to 0.653, respectively. The polymorphism information content (PIC) was between 0.029 (*Av55*) and 0.575 (*Av39*), with an average of 0.297 (Table 3). Among 72 population-by-locus tests, departure from HWE was observed at locus *Av02* in the LD population, loci *Av08* and *Av39* in the QG population after sequential Bonferroni correction (*P* < 0.004). Due to the significant *P* values of these three loci that were only present in a single population, their departures from HWE most likely reflect population-specific rather than locus-specific problems.

**Table 3:** Characteristics of 12 compound microsatellite loci developed for *A. venetum* across all the samples. Shown for each locus are the locus name, the forward (F) and reverse (R) primer sequence, allele size, repeat motif, genetic characteristics and GenBank accession number

Locus	Forward	Reverse	Size (bp)	SSR motif	*N*_A_	*H*_O_	*H*_E_	PIC	HWE	GenBank accession number
*Av02*	AAAAATGGGCAATGGTGGGC	AGGCGTAGGTGAAGAGGAGT	224	(AT)_11_	5	0.515	0.511	0.462	NS	MT737291
*Av08*	AATCAGCCACCGAGTTACCG	ACCTCCTGCAAGCTGAATCC	220	(CAT)_6_	5	0.653	0.639	0.574	NS	MT737292
*Av13*	ACGAGAAGTTGGAAACAGACCA	GTATTTGGTGTCTTCGGCGC	269	(GT)_6_	3	0.050	0.049	0.048	ND	MT737293
*Av15*	ACTCGTTGGACATGATGTGCT	GGACCTTCTCATCAGCCTCG	208	(CT)_8_	3	0.228	0.204	0.184	ND	MT737294
*Av21*	AGCAGGGGAGAAGAATGCAC	GGGTCTTGATGAGGTGAGGG	250	(ACAA)_6_	4	0.109	0.114	0.110	ND	MT737295
*Av33*	CCAAACCACACAGCTCAACG	CCAAACCACACAGCTCAACG	180	(AT)_9_	3	0.337	0.339	0.312	ND	MT737296
*Av39*	CGCTTGCTGCCTCATCATTC	CCCTCTCACACCATCCCAAC	271	(TGC)_6_	3	0.545	0.651	0.575	NS	MT737297
*Av43*	CTGCATTCCCGCAAGTAACAG	TGATGCAGCTTAGGAGGGTC	258	(AAG)_5_	2	0.356	0.294	0.250	ND	MT737298
*Av55*	GCTCCGAGAAATCCTGCTCA	GCACTGCACCCTCCTACTAC	218	(AG)_9_	3	0.030	0.030	0.029	ND	MT737299
*Av63*	GGGTTTTGCTTCTGGGCATG	GAGCCAATCCGAACCCCAC	157	(TC)_9_	2	0.327	0.301	0.254	ND	MT737300
*Av75*	TCACTAGTACCCACCACCCC	AGTGGTGGCGTTGCTATGAA	212	(GT)_6_(AT)_6_	2	0.485	0.490	0.369	NS	MT737301
*Av88*	TGCATCATGTAGGGTACACACC	GCAAGTGTTCGCTGAGTTCC	159	(AAT)_7_	4	0.396	0.487	0.400	NS	MT737302
Mean					3.25	0.336	0.342	0.297		

Notes: *N*_A_ number of alleles, *H*_*O*_ observed heterozygosity, *H*_E_ expected heterozygosity, *PIC* polymorphism information content, *HWE* Hardy-Weinberg equilibrium, *NS* Not significant for departure from HWE, *ND* Not detected for departure from HWE

### Analyses of population genetic diversity and structure

For *A. venetum*, the average estimates of genetic diversity were generally low at the population level (AR = 2.34, *H*_E_ = 0.314, *H*_O_ = 0.350). Population SY showed the lowest level of genetic diversity (AR = 1.96, *H*_E_ = 0.244, and *H*_O_ = 0.264) and population XY showed the highest (AR = 2.52, *H*_E_ = 0.363, and *H*_O_ = 0.410). The values of inbreeding (*F*_IS_) ranged from − 0.012 to − 0.138, with an average of − 0.08 (Table 4). The *F*_ST_ values of population pairs ranged from 0.032 to 0.220 (Table 5), with an overall value of 0.101, suggesting low to moderate levels of genetic differentiation across all the populations. Thirteen of the 15 pairwise comparisons were significant after sequential Bonferroni correction (Table 5).

**Table 4:** Geographic information and genetic characteristics of *A. venetum* populations based on 12 EST-SSR markers in the Yancheng coastal habitats of Jiangsu Province

Population ID	Location	Latitude (°N)	Longitude (°E)	Altitude (m)	Sample size	*N*_A_	*H*_O_	*H*_E_	AR	*F*_IS_
QG	Qianggang	32.764	120.931	4.4	14	2.25	0.369	0.332	2.19	−0.075
LD	Liangduo	32.874	120.912	9.0	24	2.25	0.303	0.293	2.09	−0.012
DF	Dafeng	32.952	120.898	−2.5	18	2.17	0.361	0.310	2.06	−0.138
XY	Xinyang	33.690	120.287	−8.0	13	2.58	0.410	0.363	2.52	−0.090
SY	Sheyang	33.877	120.432	−2.2	24	2.25	0.264	0.244	1.96	−0.061
BH	Binghai	34.008	119.789	−0.5	10	2.00	0.392	0.340	2.00	−0.102
Mean						2.25	0.350	0.314	2.34	−0.080

Notes: *N*_A_ number of alleles; *H*_O_ observed heterozygosity, *H*_E_ expected heterozygosity, *AR* allelic richness, *F*_IS_ within-population inbreeding coefficient

**Table 5:** Pairwise *F*_ST_ values among the six populations of *A. venetum*

	QG	LD	DF	XY	SY	BH
QG	0.000					
LD	0.032	0.000				
DF	**0.082**	**0.068**	0.000			
XY	**0.084**	**0.059**	**0.064**	0.000		
SY	**0.123**	**0.094**	**0.220**	**0.181**	0.000	
BH	**0.099**	**0.085**	0.061	**0.100**	**0.171**	0.000

Note: Values in bold were significantly different from zero after sequential Bonferroni correction.

**Fig. 3:**
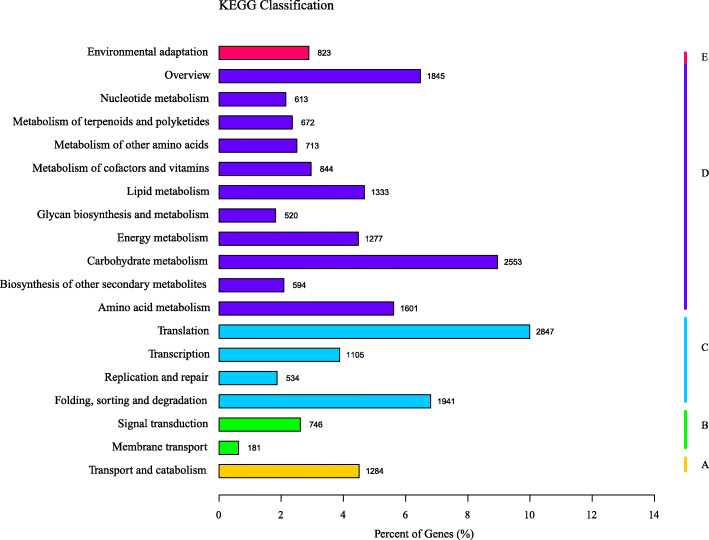
Population structure analyses of 103 *A. venetum* individuals based on the EST-SSR data. **a** Plots of the mean posterior probability [LnP(*D*)] values with genetic clusters (*K*) ranged from one to six. **b** Delta *K* values with different *K* value calculated by the method in Evanno et al. (2005). **c** Histogram of the structure analysis for the *K* value = 2 (showing the highest Δ*K*) and 3. A vertical bar represents a single individual, and ‘red’, ‘blue’ and ‘yellow’ represent cluster I-III, respectively. The x -axis corresponds to population codes and the y -axis presents the estimated membership coefficient using the Q statistic

In the structure analysis, we used LnP(*D*) and Δ*K* statistics to determine the most likely value of population genetic cluster *K*. Because the LnP(*D*) increased progressively from *K* = 1 to 6, it was difficult to determine the true number of genetic clusters (*K*). However, the Δ*K* statistic of Evanno et al. (2005) detected the highest peak at *K* = 2 (Fig. 3a, b). Figure 3c exhibits the assignment of individuals to each cluster (‘red’ and ‘blue’ represent cluster I and cluster II, respectively) when *K* = 2. The individuals that contain both colors represent the mixed origin of two gene pools. Cluster I and II were present at a high frequency (78 and 88%) (Q > 0.80) in the DF and SY populations. For the QG and XY populations, nearly half of the individuals were assigned to each cluster respectively. For the LD and BH populations, 10 to 50% of all local samples were assigned to each cluster, and the remaining 30% consisted of mixed individuals. With increased values of *K* (*K* = 3), we observed that the SY population was assigned to a separated cluster III (‘yellow’) (Fig. 3c), suggesting the potential genetic differentiation of this population from the rest of the populations in the Yancheng region.

### Historical and contemporary gene flow

The Migrate-n results revealed that the mutation-scaled effective population size (Θ) for the six *A. venetum* populations ranged from 0.0342 to 0.0438. Historical gene flow (*m*_h_) that inferred from the mutation-scaled migration rate (M) was highest from population LD to QG (*m*_h_ = 0.169), and lowest from population LD to DF (*m*_h_ = 0.041), with an average value of 0.106 across all the populations (Additional file 3: Table S1). Asymmetric gene flow was observed in two pairs of populations, with the predominant direction of gene flow occurring from population DF to LD and XY to BH. Using BayesAss software, we found that the six populations were largely composed of individuals (the average 72%) that originated from within the same site, while approximately 6% of the individuals were exchanged with each other site (Additional file 3: Table S1). Although a moderate level of gene flow was detected among the six *A. venetum* populations, the Wilcoxon signed rank test indicated that the contemporary estimates (mean *m*_c_ = 0.056) were significantly lower than the historical estimates of migration rates (mean *m*_h_ = 0.106, *P* < 0.001), suggesting a decrease in gene flow over the last few generations. The Mantel test for isolation by distance did not detect a significant correlation between genetic and geographical distance (r = 0.164, *P* = 0.251) (Additional file 4: Fig. S3).

### Changes of effective population size

Under both the stepwise mutation model (SMM) and two-phase mutation model (TPM), our Wilcoxon test detected no significant heterozygote excess for most of the populations (Table 6). For the BH population, we detected an evidence of a historical bottleneck (*P* < 0.05; Table 6). Likewise, the mode-shift test revealed that most populations showed an L-shaped distribution of alleles, suggesting the absence of a recent bottleneck. The observation of a shifted mode in the two populations (QG, BH) suggested the occurrence of bottleneck events over the last few generations (Table 6).

**Table 6:** Bottleneck analysis for six populations of *A. venetum*. *P*-values are shown for Wilcoxon’s sign-rank test under both the stepwise mutation model (SMM) and the two-phase mutation model (TPM), along with the shape of the allelic distribution inferred from the mode-shift test

Population	Wilcoxon’s sign-rank test		Mode-shift test (distribution shape) ^a^
	TPM	SMM	
QG	0.116	0.348	**shifted mode**
LD	0.348	0.688	L-shaped
DF	0.102	0.326	L-shaped
XY	0.483	0.711	L-shaped
SY	0.688	0.839	L-shaped
BH	**0.007**	**0.007**	**shifted mode**

^a^ Note that an L-shaped distribution of alleles is expected in the absence of a bottleneck, whereas a distribution with a shifted mode is expected in a population that has gone through a bottleneck

## Discussion

### Characterization of *A. venetum* transcriptome and its potential use in germplasm resources evaluation

With the decreasing costs of sequencing, the development of molecular markers that based on next-generation sequencing technology has become the most efficient method for molecular studies of non-model plants [25, 26]. Using the Illumina sequencing platform, we acquired a well-assembled transcriptome sequencing data and developed a set of novel EST-SSR markers for *A. venetum*. As an important medicinal species and source of fiber, *A. venetum* has been the subject of extensive genetics and pharmacology studies. So far, ISSR (Inter-Simple Sequence Repeat), AFLP and RAPD molecular markers have been developed for *A. venetum* [23, 24, 27]. In recent years, a number of transcriptome analyses have been conducted on *A. venetum* [20–22]. Using leaf material, we acquired a similar amount of transcriptome data (6.96 Gb) to that obtained by Chen et al. [20] (6.57 Gb) but greater numbers of unigenes (86,890 vs. 52,983) and SSRs (14,072 vs. 7579). Li et al. [22] identified 101,918 SSRs in the whole genome of *A. venetum.* Their study indicated that AT/TA and AAT/TTA accounted for the highest proportions of dinucleotide and trinucleotide repeats, respectively. In our study, we found that AG/CT (34.50%) was the most frequent motif among the dinucleotide repeats and that AAG/CTT (8.99%) was the most common trinucleotide repeat. In the study of EST-SSRs in 55 dicotyledonous species, Kumpatla and Mukhopadhyay [28] found that AG/GA/CT/TC (14.6 to 54.5% of the total SSRs observed in a species), and AAG/AGA/GAA/CTT/TTC/TCT (2.7 to 15.5%) were the predominant di- and trinucleotide SSRs. The consistent composition of EST-SSR repeats compared with most other plant species and the differences in nuclear SSRs found in *A. venetum* might suggest a transcriptional preference and conservative function of these SSR motifs in plant genomes. For instance, studies have shown that CT microsatellites in 5′ UTRs play a role in gene regulation and are involved in antisense transcription and that CTT repeats occur in 5′ UTRs and transcribed regions at a high frequency [29, 30]. *A. venetum* is widely distributed from the northwest region to the coast of the Yellow Sea in China; however, the genetic studies of *A. venetum* published to date have only focused on the arid northwest region of China. Our transcriptome data enriched the available genetic information for *A. venetum* from subhumid region and will facilitate the screening of germplasm resources, especially in coastal regions.

*A. venetum* has been used to lower blood pressure and lipemia as a traditional medicine for a long time in China, and the roasted leaves of *A. venetum* have been commercialized as a sedative and anti-ageing supplement. *A. venetum* is rich in flavonoid compounds, which provide its broad pharmacological activities [31, 32]. Flavonoids are synthesized through a long, complex pathway [33]. Identifying the regulatory mechanisms underlying flavonoid biosynthesis is essential for understanding the chemical composition or pharmacological activities of Apocynum. Previous studies have shown that *A. venetum* has a higher flavonoid content than its related species *Apocynum. hendersonii*, and some flavonoid components such as hyperoside have been identified as suitable chemical markers for the discrimination of the two species [34]. In a recent metabolome and transcriptome analyses of Apocynum, Gao et al. [21] found that the flavonoid biosynthetic pathway is responsible for a considerable proportion of the diversity between *A. venetum* and *A. hendersonii*, and identified anthocyanin as the key component that determines the phenotypic diversity of the stem and leaf color of *A. venetum* and *A. hendersonii*. In our KEGG analysis, 28,505 unigenes were clustered into 130 pathways, including the flavone and flavonol biosynthesis (ko00944), flavonoid biosynthesis (ko00941), isoflavonoid biosynthesis (ko00943), and anthocyanin biosynthesis (ko00942) pathways. Among the 88 unigenes related to these pathways, 84 unigenes have an average FPKM (Fragments per kilobase of exon per million fragments mapped) value greater than one and 24 of these were above 50. In addition, 23 out of these 88 unigenes contain SSR loci (Additional file 5: Table S2). For example, unigenes that annotated as flavonol synthase (NR ID: BAD34463.1) and flavonoid 3-O-galactosyltransferase (NR ID: BAF49284.1), both contained SSRs and exhibited an average FPKM value of 50.38 and 22.16, respectively. Although ITS and cpDNA sequences have been used to identify *A. venetum* and its related species, these sequences are conservative within the genus and only provide limited genetic information [35]. The flavonoid biosynthesis related transcripts that contain SSRs identified in our study will serve as good candidates for the development of novel molecular markers in the future, which might aid in both the species identification in the Apocynum genus and the selection of *A. venetum* germplasm resources with high bast fiber and flavonoid content.

### Low level of genetic diversity in *A. venetum* coastal populations

The disturbance of natural habitats poses a major threat to global biodiversity [36]. The ongoing process of habitat alteration generally has strong negative impacts on the species composition and genetic diversity of species [37–39]. The genetic evaluation of natural populations is an essential step in the conservation and utilization of plant resources, especially for vulnerable and threatened species. Using RAPD and AFLP markers, researchers have detected moderate to high levels of genetic diversity in natural *A. venetum* populations from the Xinjiang and Inner Mongolia regions [23, 24]. They have also found that the genetic diversity of *A. venetum* is significantly correlated with environmental factors such as precipitation, elevation and latitude. Based on EST-SSR markers, we detected a low level of genetic diversity in natural *A. venetum* populations from the coastal habitats (*H*_e_ = 0.342) (Table 4). Although EST-SSRs are expected to be more conserved and exhibit a lower rate of polymorphism than genomic SSRs [40–43], there are also studies showing that there is no significant differences between these two types of markers [44–46]. Considering the small population size of the *A. venetum* populations in the coastal region, future studies that combine the analysis of genomic SSR markers or genome-wide SNPs could better represent the real level of genetic diversity in *A. venetum* from this region.

Coastal plant species are expected to be highly vulnerable to habitat alteration, due to their high specialization to coastal environments [6]. Through nuclear SSR analysis, a low level of genetic diversity has been observed in coastal herb, shrub and tree species [47–49]. Peng et al. [50] investigated the genetic diversity of nine wild *A. venetum* populations from eight provinces of China using RAPD markers, and their study showed that populations from coastal regions such as Jiangsu and Jilin exhibit lower genetic diversity than those from the arid northwest regions such as Xinjiang, Inner Mongolia and Ningxia. In a comparative study of *Fraxinus angustifolia* Vahl populations from the Continental region and the Mediterranean region, Temunović et al. [51] detected a significantly lower genetic diversity and higher population divergence in the latter region, revealing the influence of environmental heterogeneity in shaping the genetic variation between divergent habitats. Therefore, coastal *A. venetum* populations might represent a divergent ecotype, and environmental factors may cause differences in genetic diversity between arid and subhumid regions. By including environmental data in our future analyses, we could further test whether the differences are correlated with diverse natural conditions (subhumid vs. arid areas). Another plausible explanation for this low level of genetic diversity is that *A. venetum* populations in the coastal region have experienced severe reductions in population size, leading to the loss of genetic diversity and increased susceptibility. Using Bottleneck software, we found that two populations (QG and BH) showed distortions (mode-shifts) in their allele frequency distributions, suggesting recent bottleneck events, and Wilcoxon’s test also revealed significant heterozygote excess in the BH population (*P* < 0.05; Table 6). According to our field observations, for populations such as BH and SY, situated along the highway road, the natural habitat is severely being disturbed. In addition, the population size of most populations is quite small (*N* < 30). Based on scaled effective population sizes Θ (4*N*_e_μ), the estimated *N*_e_ of the *A. venetum* populations in the Yancheng region is approximately 10–11 individuals. According to a genetic model predicting the proportion of initial heterozygosity retained per generation [1-(1/2*N*_e_)] [52], *A. venetum* populations would be expected to lose 4.5% of their heterozygosity per generation. Therefore, with declining genetic diversity and continuing demographic changes over time, the *A. venetum* populations in the Yancheng coastal region are of potential concern in terms of decreases in individual fitness and population viability, and an increased risk of extinction in the future.

### Moderate level of gene connection with a decreasing trend in *A. venetum* coastal populations

Gene flow is crucial for population resilience and persistence following habitat disturbances and environmental changes. This process may provide a source of recruitment and maintain genetic diversity by introducing adaptive alleles from other populations [53–55]. In this study, we found little evidence of strong genetic structuring in the *A. venetum* populations across the Yancheng coastal region. Genetic structure analysis grouped all of the sampled populations into two clusters, and in the majority of populations, a number of admixed plants existed (Fig. 3c). Negative multilocus *F*_IS_ values (mean value of − 0.08) were found for *A. venetum*, indicating an absence of inbreeding in the coastal populations of this species (Table 4). In accordance with this, contemporary migration rates among *A. venetum* coastal populations were found to be moderate (mean *m*_c_ = 0.056) (Additional file 3: Table S1). Together, these results indicate that population connectivity in *A. venetum* coastal populations has not yet been greatly disturbed yet. In general, life-history traits of plant species such as their pollen and seed dispersal mode, often affect the genetic connection between populations [55, 56]. The study of pollination biology has shown that *Apis mellifera* and Ichneumon sp. are the main pollinators of *A. venetum* [57]. Due to the limited foraging distances of these pollinators, gene dispersal through pollen alone seems insufficient to maintain the species’ population connectivity across the coastal region. The seeds of *A. venetum* are less than 1 mm in size and are covered with pappus-like hair, which enables an anemochorous dispersion [58, 59]. The high seed dispersal capabilities of *A. venetum* may play a key role in maintaining the moderate levels of ongoing gene flow and population connectivity across the coastal region. Although studies have shown that plants relying on wind for pollination or seed dispersal may be subject to less negative effects of habitat alteration on their genetic diversity or population connection [55, 56], we still detected a decrease in gene flow over the last few generations compared to the historical level of migration rates (mean *m*_h_ = 0.106). Under the persistent pressures of anthropogenic and climate disturbances, whether natural *A. venetum* populations, especially those with low genetic diversity and a small population size, have a sufficient capacity to survive following disturbance or adapt to future environmental change still requires long-term monitoring.

## Conclusion

In this study, we acquired a high-quality transcriptome from *A. venetum* leaves by using the Illumina sequencing platform and successfully developed twelve polymorphic EST-SSR markers in *A. venetum*. Using these informative makers, we detected a low level of genetic diversity and bottleneck events in *A. venetum* populations across their natural distribution in the Yancheng coastal region of Jiangsu Province. Although population connectivity between *A. venetum* populations has been maintained by the high seed dispersal ability of the species, considering the ongoing anthropogenic activities, long-term monitoring and conservation strategies should be implemented to better protect these small populations. The conservation of available genetic diversity is essential to enable the continued utilization of this economically important plant. Based on the results of this study, we suggested that populations that have undergone bottlenecks (e.g., population BH and QG) and those with a high level of genetic diversity (population XY), should be given conservation priority. In addition, there are not enough comparative data available on the extent of genetic diversity in *A. venetum* across its global biogeographical ranges, and further studies combining samples from arid regions and more markers should be conducted to gain a more precise understanding of the genetic diversity, population structure, and evolutionary history of this important plant species.

## Methods

### Sample collection and DNA and RNA extraction

**Fig. 4:**
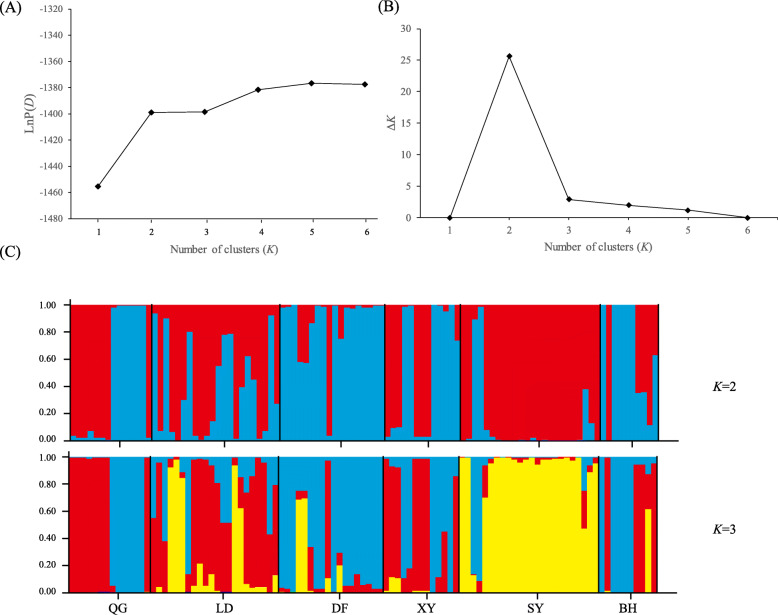
Sample localities of *Apocynum venetum* in the Yancheng coastal habitats of Jiangsu Province. Population code is listed in Table 4

A total of 103 leaf samples of *A. venetum* were collected from 10 to 24 individuals in six wild populations, representing most of the distribution area of this species in Jiangsu Province (Fig. 4). Detailed information for all the populations is listed in Table 4. *A. venetum* is mainly distributed along the riversides or roadsides, and permission was not necessary to collect these samples. The collection of plant material complied with national guidelines. All sampled leaves were immediately dried in silica gel. The materials were identified by Jiangsu Province and Chinese Academy of Sciences, China. We used QIAGEN Plant DNA kit (Gaithersburg, MD) to extract the total genomic DNA according to the manufacturer’s protocol. Besides, fresh leaves of three individuals from the LD population were immediately frozen in liquid nitrogen and stored at − 70 °C for total RNA extraction. Total RNA from these individuals was extracted using the QIAGEN RNeasy Plant Mini Kit following the manufacturer’s procedures (Chatsworth, CA). Then the quantified total RNAs were sent to Novogene Bioinformatics Institute (Beijing, China) for further processing.

### cDNA library construction and transcriptome sequencing

First, the RNA concentration and integrity of the samples were measured using the Qubit RNA Assay Kit in a Qubit 2.0 Fluorometer (Life Technologies, Carlsbad, CA, USA) and the RNA Nano 6000 Assay Kit with the Agilent Bioanalyzer 2100 system (Agilent Technologies, Santa Clara, CA, USA). Then, for each sample, cDNA library were construction and sequenced by using equal amounts of qualified RNA according to the standard procedures of Novogene Bioinformatics Institute (Beijing, China). The NEBNext® Ultra™ RNA Library Prep Kit for Illumina® (NEB, USA) were used to generate sequencing libraries. After purification and quality assessment, the three library preparations were sequenced on the Illumina HiSeq platform (Illumina, USA).

### Transcriptome assembly and functional annotation

Raw RNA-seq reads were processed using in-house Perl scripts to remove reads containing adapters and ploy-N sequences (greater than 5%) and reads with more than 20% low-quality bases (quality scores < 10). Then, we used Trinity software [60] with the default parameters to assemble the high-quality clean data. All unigenes were queried against the NCBI Nr (non-redundant protein) and Nt (non-redundant nucleotide) databases; the SwissProt protein database (http://www.expasy.ch/sprot); Pfam (Protein family database); the KOG (Clusters of Orthologous Groups of proteins) database and KO (KEGG Orthology) database. Gene ontology (GO) annotation [61] of the unigenes was performed using BLAST2go [62]. The Kyoto Encyclopedia of Genes and Genomes (KEGG) pathways (https://www.genome.jp/kegg/) were determined with an E-value cut-off of 1e-5. The WEGO [63] were used to plot the distributions of level-2 GO terms with functional classification.

### Identification of EST-SSRs

The MicroSAtellite (MISA) program [64] was used to detect transcripts containing EST-SSRs. A minimum repeat number of six for dinucleotide motifs and five for tri-, tetra-, penta-, and hexanucleotide motifs were set as detection criteria. The sequences containing SSRs were submitted to Primer premier 5.0 software (Premier Biosoft International, Palo Alto, CA, USA) to design primers. The parameter settings were as follows: product size ranging from 100 to 300 bp; primer length ranging from 18 to 25 bp; GC content between 40 and 60% and the annealing temperature between 55 and 65 °C [65].

PCR amplification was carried out in a 15 μL total reaction volume containing 20–40 ng genomic DNA, 7.5 μl of 2 × Taq PCR MasterMix (Tiangen, Beijing, China) and 0.3 μM of each primer. The PCR procedure included an initial denaturation for 3 min at 94 °C, followed by 35 cycles of 30 s at 94 °C, 30 s at optimal annealing temperature for each locus, and 15 s at 72 °C, followed by a final extension of 5 min at 72 °C. The PCR products were checked using silver-stained nondenaturing polyacrylamide gels. Then the optimized SSR primers were further labelled with 6-FAM or HEX fluorescein dye (Sangon Biotech, Shanghai, China). After PCR amplification, allele identification and genotyping were performed with GeneMarker version 2.2.0 (SoftGenetics, State College, Pennsylvania, USA).

### Genetic diversity analyses

The number of alleles (*N*_A_), polymorphism information content (PIC), expected heterozygosity (*H*_E_) and observed heterozygosity (*H*_O_) of each EST-SSR locus were estimated with Cervus 2.0 [66]. Hardy-Weinberg equilibrium (HWE) and linkage disequilibrium (LD) tests were performed using Genepop version 4.2 with a Bonferroni correction [67]. Null allele frequencies, stuttering, and large allele dropout were detected using the Micro-Checker version 2.2.3 [68] program. For each population of *A*. *venetum*, the number of alleles (*N*_A_), allelic richness (AR), expected heterozygosity (*H*_E_), observed heterozygosity (*H*_O_), inbreeding coefficient (*F*_IS_) and differentiation among populations (*F*_ST_) were calculated using Fstat version 2.9.3.2 [69].

### Population genetic structure, gene flow and demographic analyses

Population structure was investigated by using the Sructure 2.3.4 program [70] implementing a model based Bayesian approach. The value of genetic clusters (*K*) was set from 1 to 6, assuming an admixture model and independent allele frequencies. Ten independent runs were conducted for each *K* with a burn-in of 10,000 and 100,000 Markov Chain Monte Carlo replicates. The most possible *K* value was chosen by calculating Δ*K* [71] in Structure Harvester [72].

Contemporary inter-population migration between *A. venetum* populations was estimated using BayesAss version 1.3 [73]. The delta values for allele frequencies, migration rates, and inbreeding coefficients were adjusted accordingly to ensure that the acceptance rates fell between 40 and 60% [73]. We performed the software for 10^7^ iterations with a burn-in of 10^6^ generations. Ten replicate runs were conducted with a different initial seed. We assessed the model convergence by comparing the posterior probability densities of parameter estimates across these ten runs. The results presented were from the best-fit run. We also estimated historical gene flow using a coalescent-based Bayesian method implemented in the program Migrate-n version 3.6 [74]. The process used the Brownian motion approximation as the mutation model following 10^7^ iterations with a burn-in of 10^5^. The static heating scheme was used with four chains at different temperatures (1.0, 1.5, 3.0, and 100,000.0) [75]. Two parameters, scaled effective population sizes Θ (4*N*_e_μ, where *N*_e_ is effective population size, μ is the average mutation rate of microsatellites as 10^− 3^ per generation) and scaled immigration rates M (*m*_h_/μ, where *m*_h_ is historical migration rate) between pairs of populations over around 4*N*_e_ generations were estimated simultaneously. The number of immigrants per generation Nm was estimated by the equation Nm = ΘM/4. A Wilcoxon signed-rank test was conducted to compare historical and contemporary gene flow estimates among the *A. venetum* populations.

To test for isolation-by-distance (IBD), the correlation of pairwise geographical distance (log geographical distance in km) and genetic distance (*F*_ST_/1-*F*_ST_) values was evaluated. Statistical significance was tested with 1000 permutations of the Mantel test via the R package Vegan [76]. Wilcoxon’s signed rank test and the mode-shift test in Bottleneck version 1.2.02 [77] were used to determine whether the *A. venetum* populations in the Yancheng coastal region have undergone significant reductions in the effective population size (*N*_e_). The first methodology compares the heterozygosity expected (*H*_E_) at Hardy-Weinberg equilibrium with the heterozygosity expected at mutation-drift equilibrium (*H*_eq_) [78], which is suitable for detecting bottlenecks occurring in the last 2-4*N*_e_ generations. The second methodology is based on the allele frequency distribution, which is more appropriate for detecting population declines that have occurred more recently (approximately the last few dozen generations) [79, 80]. For population that has not experienced bottleneck event, a large proportion of alleles at a low frequency and a smaller proportion of alleles at intermediate frequencies distribution (L-shape distribution) presents. While in bottlenecked populations, a shifted mode of allele frequency distribution would be detected. We performed 10,000 simulations under both the stepwise mutation model (SMM) and the two-phase model (TPM) with 95% single-step mutations and 5% multistep mutations for each *A. venetum* population. *P*-values were assessed for statistical significance at the 0.05 level.

## Supplementary information

**Additional file 1: Fig. S1.** Length distribution of assembled unigenes (A) and the number of reads mapped to each unigene (B) that generated from *A. venetum* transcriptome.

**Additional file 2: Fig. S2.** KOG classification of assembled unigenes of *A. venetum*.

**Additional file 3: Table S1.** Migrate-n results of historical gene flow and BayesAss results of contemporary gene flow with 95% confidence among the *Apocynum venetum* populations.

**Additional file 4: Fig. S3.** Correlation between genetic differentiation and log geographical distance for the *A. venetum* populations in Jiangsu Province.

**Additional file 5 **Table S2 The SSR type, expression level and KEGG annotation of 88 flavonoid biosynthesis related genes in *A. venetum*.

## Data Availability

Raw sequencing data used for EST-SSR marker development are available in NCBI SRA: PRJNA614557 (https://www.ncbi.nlm.nih.gov/sra/PRJNA614557). Other datasets supporting the conclusions of this article are included within the article and its additional files.

## Abbreviations

AR: Allelic richness
EST-SSR: Expressed sequence tag-derived simple sequence repeat markers
*F*_IS_: Inbreeding coefficient
*F*_ST_: Differentiation among populations
GO: Gene ontology
*H*_E_: The expected heterozygosity
*H*_O_: Observed heterozygosity
HWE: Hardy-Weinberg equilibrium
KO: KEGG Ortholog database
KOG: Clusters of Orthologous Groups of proteins
*N*_e_: Effective population size
Nr: NCBI non-redundant protein database
Nt: NCBI non-redundant nucleotide database
Pfam: Protein family database
