# Supplementary figures and images for: De novo transcriptome assembly and population genetic analyses of an important coastal shrub, Apocynum venetum L
Source: BMC Plant Biol. 2020 Sep 3;20:408. doi: 10.1186/s12870-020-02626-7 (PMC7470449; doi:10.1186/s12870-020-02626-7)

(A)

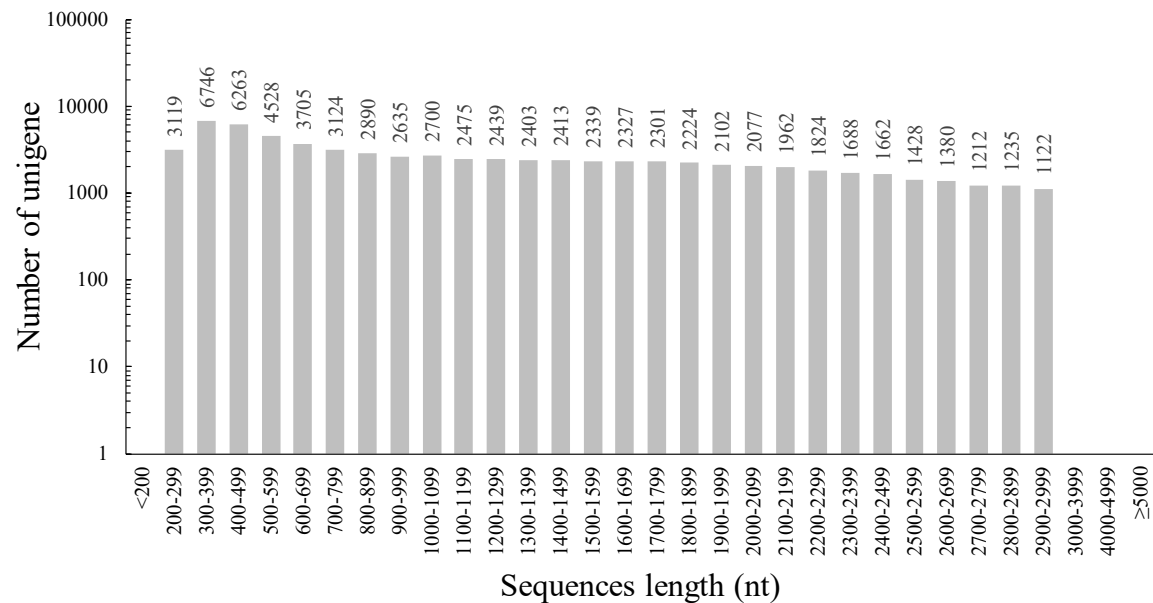

(B)

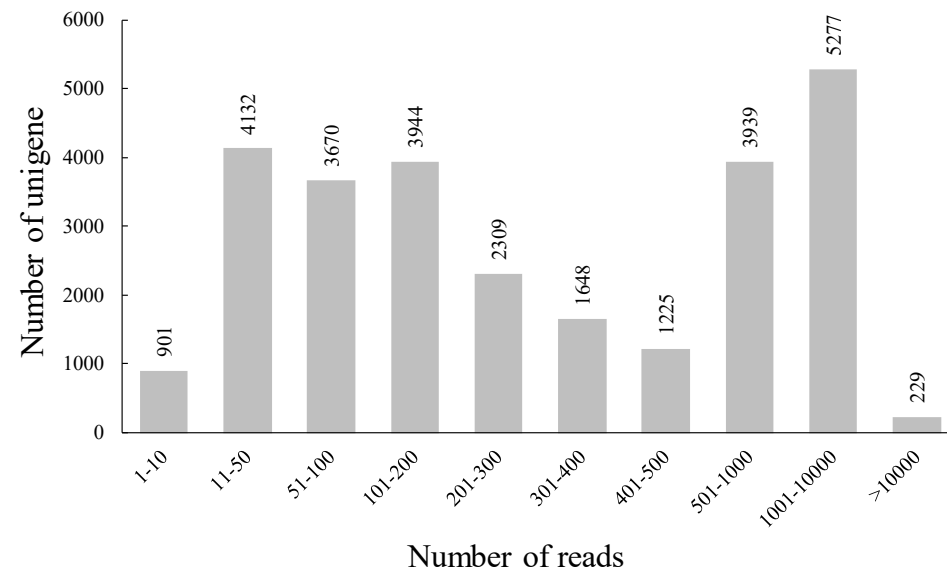

Supplement: Supplementary file 1 — Additional file 1: Fig. S1. Length distribution of assembled unigenes (A) and the number of reads mapped to each unigene (B) that generated from A. venetum transcriptome. [file 12870_2020_2626_MOESM1_ESM.pdf]

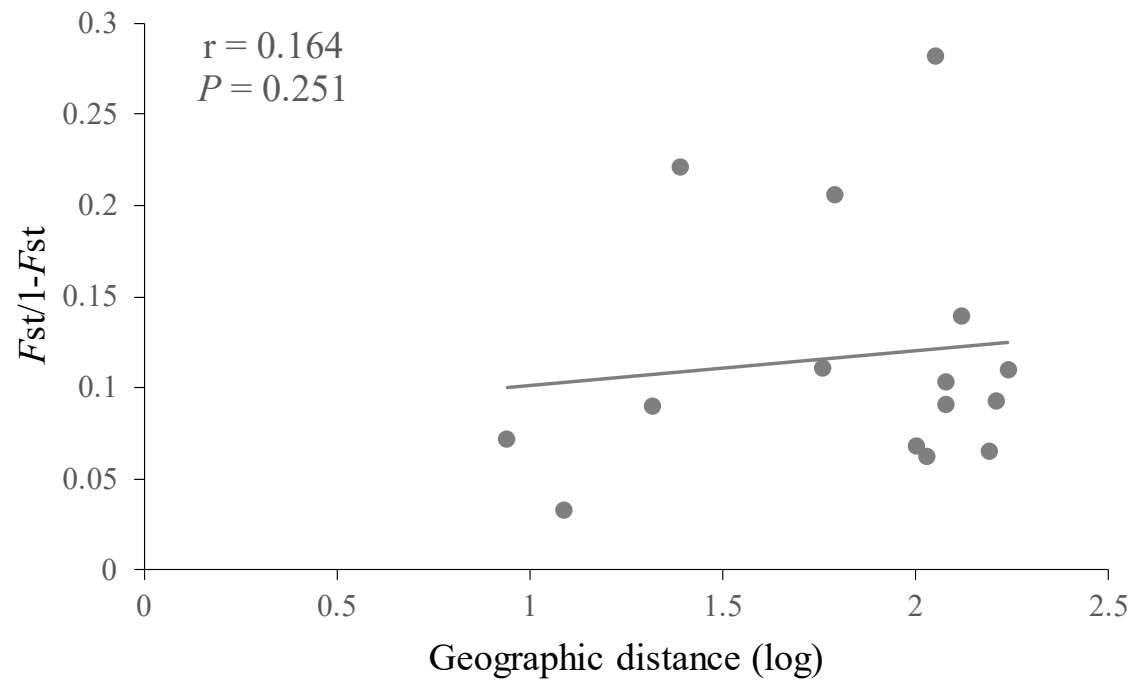

Supplement: Supplementary file 4 — Additional file 4: Fig. S3. Correlation between genetic differentiation and log geographical distance for the A. venetum populations in Jiangsu Province. [file 12870_2020_2626_MOESM4_ESM.pdf]
