# Supplementary material for: De novo transcriptome assembly and population genetic analyses of an important coastal shrub, Apocynum venetum L
Source: BMC Plant Biol. 2020 Sep 3;20:408. doi: 10.1186/s12870-020-02626-7 (PMC7470449; doi:10.1186/s12870-020-02626-7)
Supplement: Supplementary file 2 — Additional file 2: Fig. S2. KOG classification of assembled unigenes of A. venetum. [file 12870_2020_2626_MOESM2_ESM.pdf]

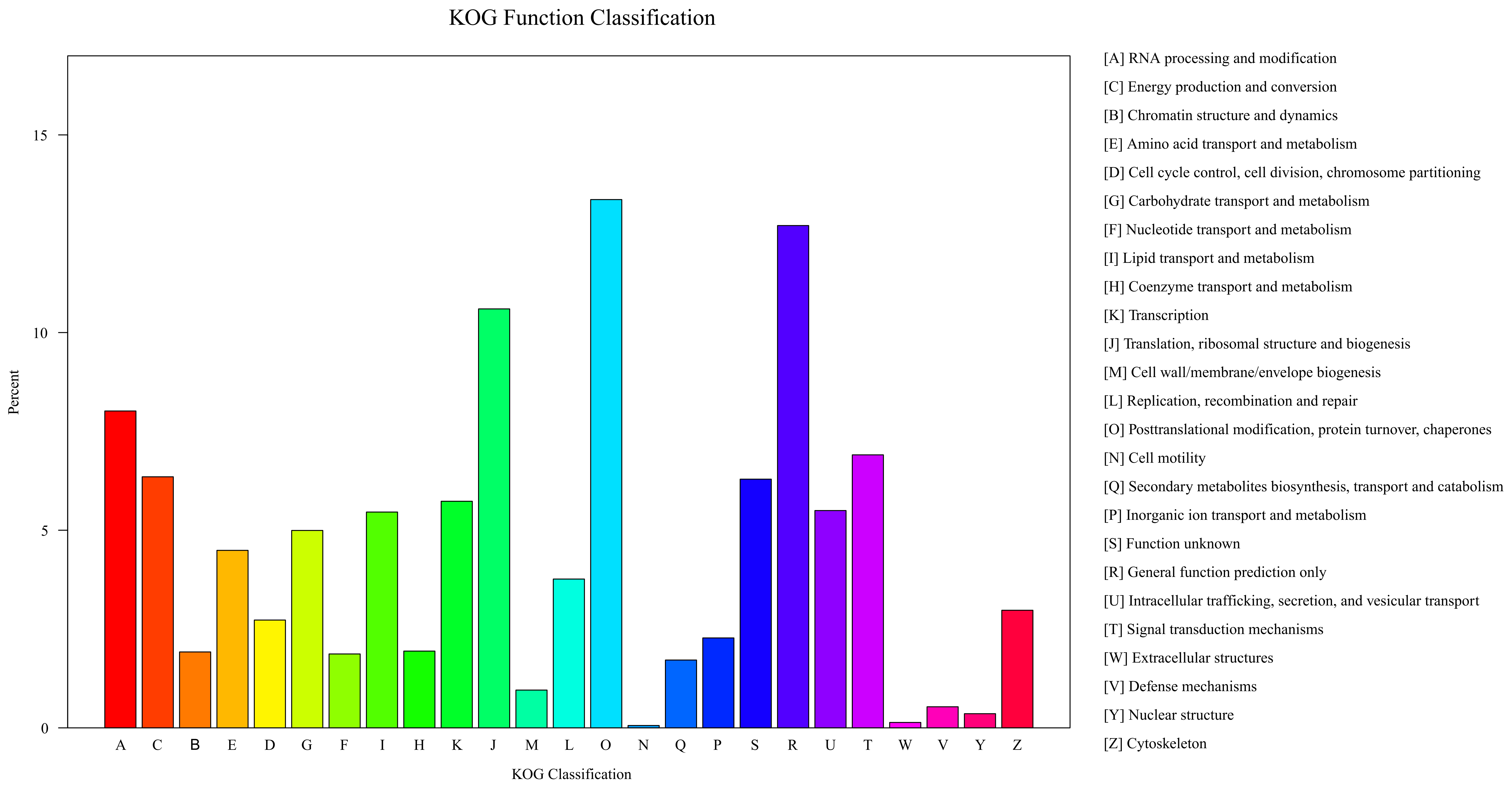
KOG Function Classification
15
10
5
0
Percent
A
C
B
E
D
G
F
I
H
K
J
M
L
O
N
Q
P
S
R
U
T
W
V
Y
Z
KOG Classification
[A] RNA processing and modification
[C] Energy production and conversion
[B] Chromatin structure and dynamics
[E] Amino acid transport and metabolism
[D] Cell cycle control, cell division, chromosome partitioning
[G] Carbohydrate transport and metabolism
[F] Nucleotide transport and metabolism
[I] Lipid transport and metabolism
[H] Coenzyme transport and metabolism
[K] Transcription
[J] Translation, ribosomal structure and biogenesis
[M] Cell wall/membrane/envelope biogenesis
[L] Replication, recombination and repair
[O] Posttranslational modification, protein turnover, chaperones
[N] Cell motility
[Q] Secondary metabolites biosynthesis, transport and catabolism
[P] Inorganic ion transport and metabolism
[S] Function unknown
[R] General function prediction only
[U] Intracellular trafficking, secretion, and vesicular transport
[T] Signal transduction mechanisms
[W] Extracellular structures
[V] Defense mechanisms
[Y] Nuclear structure
[Z] Cytoskeleton
