# Supplementary material for: De novo transcriptome assembly and population genetic analyses of an important coastal shrub, Apocynum venetum L
Source: BMC Plant Biol. 2020 Sep 3;20:408. doi: 10.1186/s12870-020-02626-7 (PMC7470449; doi:10.1186/s12870-020-02626-7)
Supplement: Supplementary file 3 — Additional file 3: Table S1. Migrate-n results of historical gene flow and BayesAss results of contemporary gene flow with 95% confidence among the Apocynum venetum populations. [file 12870_2020_2626_MOESM3_ESM.doc]

Table S1 Migrate-n results of historical gene flow and BayesAss results of contemporary gene flow with 95% confidence among the *Apocynum venetum* populations.

|  | Migrate-n | | | | Bayesass | |
| --- | --- | --- | --- | --- | --- | --- |
| Populations | M | Nm | *m*_h_ | 95% Confidence | *m*_c_ | 95% Confidence |
| 2→1 | 169.247 | 1.896 | 0.169 | 0.095-0.241 | 0.055 | 0.036-0.146 |
| 3→1 | 115.701 | 1.296 | 0.116 | 0.045-0.193 | 0.057 | 0.037-0.152 |
| 4→1 | 117.649 | 1.318 | 0.118 | 0.046-0.181 | 0.055 | 0.034-0.144 |
| 5→1 | 108.959 | 1.221 | 0.109 | 0.051-0.129 | 0.051 | 0.036-0.137 |
| 6→1 | 97.377 | 1.091 | 0.097 | 0.024-0.221 | 0.050 | 0.034-0.133 |
| 1→2 | 105.135 | 1.232 | 0.105 | 0.056-0.142 | 0.066 | 0.035-0.167 |
| 3→2 | 127.027 | 1.488 | 0.127* | 0.053-0.091 | 0.057 | 0.036-0.150 |
| 4→2 | 129.879 | 1.522 | 0.13 | 0.084-0.177 | 0.055 | 0.036-0.145 |
| 5→2 | 91.196 | 1.068 | 0.091 | 0.037-0.155 | 0.051 | 0.035-0.136 |
| 6→2 | 87.588 | 1.026 | 0.088 | 0.044-0.125 | 0.050 | 0.034-0.134 |
| 1→3 | 75.122 | 0.849 | 0.075 | 0.030-0.133 | 0.067 | 0.036-0.170 |
| 2→3 | 41.248 | 0.466 | 0.041* | 0.013-0.072 | 0.054 | 0.036-0.143 |
| 4→3 | 78.96 | 0.892 | 0.079 | 0.032-0.131 | 0.056 | 0.037-0.149 |
| 5→3 | 55.924 | 0.632 | 0.056 | 0.013-0.117 | 0.051 | 0.035-0.137 |
| 6→3 | 105.884 | 1.196 | 0.106 | 0.046-0.173 | 0.050 | 0.034-0.134 |
| 1→4 | 122.593 | 1.361 | 0.123 | 0.057-0.191 | 0.067 | 0.035-0.169 |
| 2→4 | 136.016 | 1.51 | 0.136 | 0.046-0.226 | 0.054 | 0.036-0.144 |
| 3→4 | 131.534 | 1.46 | 0.132 | 0.047-0.205 | 0.058 | 0.037-0.154 |
| 5→4 | 105.235 | 1.168 | 0.105 | 0.045-0.171 | 0.052 | 0.035-0.139 |
| 6→4 | 60.004 | 0.666 | 0.060* | 0.017-0.119 | 0.052 | 0.034-0.138 |
| 1→5 | 76.702 | 0.892 | 0.077 | 0.015-0.167 | 0.067 | 0.035-0.169 |
| 2→5 | 76.349 | 0.888 | 0.076 | 0.016-0.149 | 0.055 | 0.037-0.147 |
| 3→5 | 93.377 | 1.086 | 0.093 | 0.041-0.155 | 0.057 | 0.036-0.150 |
| 4→5 | 90.759 | 1.056 | 0.091 | 0.038-0.144 | 0.055 | 0.038-0.148 |
| 6→5 | 73.488 | 0.855 | 0.073 | 0.030-0.107 | 0.050 | 0.034-0.133 |
| 1→6 | 141.265 | 1.493 | 0.141 | 0.051-0.220 | 0.066 | 0.034-0.166 |
| 2→6 | 140.152 | 1.481 | 0.14 | 0.074-0.203 | 0.055 | 0.036-0.145 |
| 3→6 | 169.743 | 1.794 | 0.17 | 0.108-0.220 | 0.057 | 0.036-0.151 |
| 4→6 | 127.464 | 1.347 | 0.127* | 0.071-0.187 | 0.056 | 0.037-0.149 |
| 5→6 | 113.616 | 1.201 | 0.114 | 0.049-0.174 | 0.051 | 0.035-0.137 |
| Mean |  | 1.182 | 0.106 | 0.046-0.164 | 0.056 | 0.036-0.147 |

M, mutation scaled migration rate

Nm (ΘM/4, where Θ is scaled effective population sizes), the number of migrants per generation

*m*_h_ (Mμ, where μ is mutation rate), historical migration rate

*m*_c_, contemporary migration rate

* asymmetric gene flow

1 QG

2 LD

3 DF

4 XY

5 SY

6 BH
